# How to assess ventricular function by fetal echocardiography: expert guidance from the Fetal Heart Society

**DOI:** 10.1002/uog.70276

**Published:** 2026-07-02

**Authors:** S. R. Patel, N. Madan, L. Eckersley, Z. Roytman, M. Perez, L. Gindes, L. K. Hornberger

**Affiliations:** ^1^ Division of Cardiology, Ann and Robert H. Lurie Children's Hospital of Chicago Northwestern University Feinberg School of Medicine Chicago IL USA; ^2^ Division of Cardiology, Children's Mercy Hospital University of Missouri‐Kansas City School of Medicine Kansas City MO USA; ^3^ Division of Cardiology, Department of Pediatrics, Women's & Children's Health Research Institute University of Alberta, Stollery Children's Hospital Edmonton AB Canada; ^4^ Division of Maternal‐Fetal Medicine Scripps Health San Diego CA USA; ^5^ Ultrasound Unit, Department of Obstetrics and Gynecology Edith Wolfson Medical Center Holon Israel; ^6^ Faculty of Medical and Health Sciences Tel‐Aviv University Tel‐Aviv Israel

**Keywords:** Doppler, fetal echocardiography, hydrops fetalis, M‐mode, speckle tracking, ventricular function

How to… Practical advice on imaging‐based techniques and investigations with accompanying videoclips online

## INTRODUCTION

Assessment of ventricular function is an integral component of fetal cardiac evaluation^1^, ^2^. In most routine examinations, it is sufficient to confirm the absence of cardiomegaly or atrioventricular (AV) valve regurgitation, and to perform a qualitative assessment of ventricular systolic function and Doppler evaluation of ventricular filling and central venous pressure. However, more comprehensive quantitative measures are particularly important in fetuses at risk of ventricular dysfunction. For example, in twin‐to‐twin transfusion syndrome, the recipient twin may start to show features suggestive of ventricular dysfunction, sometimes even before changes in the amniotic fluid volume or growth discrepancy with the cotwin become evident. Additionally, conditions that affect the myocardium itself, such as viral myocarditis, cardiomyopathies or immune‐mediated myocarditis (e.g. SSA/SSB antibodies), can cause poor ventricular function. Conditions that can increase cardiac afterload, such as premature ductal constriction, valve stenosis or placental pathologies, can adversely affect ventricular function. Moreover, ventricular function can also be affected in fetuses with arrhythmia or high‐output cardiac lesions.

Fetal cardiovascular physiology is uniquely characterized by a shared preload and combined output of the right (RV) and left (LV) ventricles, a low‐resistance afterload provided by the placenta, and a circulation that demands low ventricular filling pressures. Additionally, abrupt hemodynamic changes at birth resulting from closure of the fetal shunts and placental separation may precipitate hemodynamic instability in fetuses with ventricular dysfunction. Therefore, accurate prenatal assessment of ventricular function is essential for planning surveillance, delivery and resource needs. This How To article outlines a step‐by‐step approach for evaluating fetal ventricular function using echocardiography, supported by figures and videos. A summary of the different measures of fetal cardiac function is provided in Table 1.

**Table 1:** Summary of echocardiographic parameters used to assess fetal cardiac function

Method	Images to obtain	What to assess	Calculation	Normal observations	Indicators of dysfunction
Qualitative	2D ultrasound cine loops in apical four‐chamber and ventricular short‐axis views.	Assess global contractility of the heart and contractility (squeeze) and symmetry between ventricles, wall motion and changes in cavity size.	NA	Ventricles contract synchronously (rhythm) and symmetrically, with normal pumping function.	Abnormal regional or global wall motion abnormalities.
SF (%)	2D ultrasound cine loops in short‐axis view at level of papillary muscles or in four‐chamber view with ventricular walls axial to ultrasound beam.	M‐mode or 2D measures of end‐diastolic dimensions, wall thickness and end‐systolic dimensions.	LV‐SF (%) =$\frac{\text{LVEDD}\;-\;\text{LVESD}}{\text{LVEDD}}\times 100$ RV‐SF (%) = $\frac{\text{RVEDD}\;-\;\text{RVESD}}{\text{RVEDD}}\times 100$	GA‐specific values are published^3^. Typically, LV‐SF > 28% and RV‐SF > 24%.	Abnormal values for GA, chamber dilation, wall or septal hypertrophy.
MAPSE/TAPSE	Obtain apical four‐chamber view. Place M‐mode cursor from ventricular apex through lateral mitral and tricuspid valve annuli.	Measure excursion from lowest to highest point.	NA	GA‐specific values are published^4^. Typical ranges: MAPSE, 2.87–5.56 mm; TAPSE, 2.34–4.21 mm.	Abnormal values for GA.
EF (%)	2D ultrasound cine loops in apical four‐chamber view. Ventricular apex at center of image and AVV annular plane in horizontal orientation.	Software‐derived ventricular EDV and ESV using summation of LV ‘disks’ and assuming an ellipsoid LV.	LV‐EF (%) = $\frac{\text{LV}‐\text{EDV}\;-\;\text{LV}‐\text{ESV}}{\text{LV}‐\text{EDV}}\times 100$	Typically, LV‐EF > 55% is considered normal^6^.	LV‐EF ≤ 55% is considered abnormal.
FAC (%)	2D ultrasound cine loops in apical four‐chamber view. Ventricular apex at center of image and AVV annular plane in horizontal orientation.	Trace ventricular end‐diastolic area and end‐systolic area along endomyocardial border.	LV‐FAC (%) = $\frac{\text{LVEDA}\;-\;\text{LVESA}}{\text{LVEDA}}\times 100$ RV‐FAC (%) =$\frac{\text{RVEDA}\;-\;\text{RVESA}}{\text{RVEDA}}\times 100$	GA‐specific values are published^8^, ^9^. Typically, LV‐FAC > 24% and RV‐FAC > 20%.	Abnormal values for GA.
CCO (mL/kg/min)	Obtain aortic and pulmonary valve annular dimensions in long‐axis plane. PWD across aortic valve and pulmonary valve leaflet tips, < 20° angle of insonation (as close to 0° as possible).	Measure aortic and pulmonary annular diameter. Measure HR. Trace PWD envelope to obtain VTI. Obtain EFW.	LCO (mL/min) = $\pi \times {\left(\frac{\mathrm{AoV}\;d}{2}\right)}^{2}\times \mathrm{VTI}\times \text{FHR}$ RCO (mL/min) = $\pi \times {\left(\frac{\mathrm{PV}\;d}{2}\right)}^{2}\times \mathrm{VTI}\times \text{FHR}$ CCO (mL/kg/min) = (LCO + RCO)/EFW	GA‐specific values are published^15^, ^16^. CCO values typically increase through gestation.	Abnormal values for GA.
MPI	LV‐MPI: PWD cursor placed within LV cavity to obtain MV and LVOT simultaneously. RV‐MPI: PWD assessment across TV and PV in succession.	Measure inflow ET.	LV‐MPI = $\frac{\text{ICT}+\text{IRT}}{\text{LV}‐\text{ET}}$ RV‐MPI = $\frac{\text{TV}‐\text{CO}-\text{RV}‐\text{ET}}{\text{RV}‐\text{ET}}$	GA‐specific values are published using both spectral^20^, ^21^ and tissue Doppler^22^ techniques. Typically, MPI < 0.4 is considered normal. MPI values typically increase through gestation.	Abnormal values for GA.
	MPI using spectral tissue Doppler: obtain apical four‐chamber view. Place spectral tissue Doppler cursor with sample volume on lateral aspect of TV and lateral aspect of MV.	Use E′, A′ and S′ signals to measure ICT, ET and IRT.	MPI = $\frac{\mathrm{ICT}+\mathrm{IRT}}{\mathrm{ET}}$		
Diastolic function	Obtain apical four‐chamber view. Place PWD at MV and TV leaflet tips.	MV and TV E‐ and A‐wave velocities and E/A ratio, which vary by GA^3^.	E/A ratio, inflow duration	Biphasic MV and TV inflow Doppler.	Monophasic MV and TV inflow Doppler or E > A in restrictive physiology and cardiac compression.
	PWD cursor placed 1–2 mm into pulmonary vein from the junction with LA.	S‐ and D‐wave velocities.	NA	Continuous flow from pulmonary vein into LA through whole cardiac cycle with a systolic and a diastolic peak.	A‐wave reversal.
	PWD in ductus venosus with sagittal insonation.	S‐, D‐, A‐wave velocities.	S:A ratio	Continuous forward flow in triphasic pattern.	A‐wave absence or reversal.
	PWD in umbilical vein in free cord loop.	Non‐pulsatile (normal) *vs* pulsatile flow pattern, waveform appearance.	NA	Even flow without any fluctuation.	Presence of pulsations defined as > 15% reduction from baseline velocity.

2D, two‐dimensional; A, atrial contraction; AoV d, aortic valve diameter; AVV, atrioventricular valve; CCO, combined cardiac output; CO, cardiac output; D, diastole; E, early diastole; EDA, end‐diastolic area; EDD, end‐diastolic diameter; EDV, end‐diastolic volume; EF, ejection fraction; EFW, estimated fetal weight; ESA, end‐systolic area; ESD, end‐systolic diameter; ESV, end‐systolic volume; ET, ejection time; FAC, fractional area change; FHR, fetal heart rate; GA, gestational age; HR, heart rate; ICT, isovolumetric contraction time; IRT, isovolumetric relaxation time; LA, left atrium; LCO, left cardiac output; LV, left ventricle; LVOT, left ventricular outflow tract; MAPSE, mitral annular plane systolic excursion; MPI, myocardial performance index; MV, mitral valve; NA, not applicable; PV, pulmonary valve; PV d, pulmonary valve diameter; PWD, pulsed‐wave Doppler; RCO, right cardiac output; RV, right ventricle; S, systole; SF, shortening fraction; TAPSE, tricuspid annular plane systolic excursion; TV, tricuspid valve; VTI, velocity time integral.

## PRACTICAL POINTS

### Assessing ventricular systolic function

#### Qualitative and quantitative assessment of systolic function

Current fetal echocardiography guidelines^1^, ^2^ recommend qualitative assessment of biventricular function at every examination. Global systolic function may be appraised subjectively based on the visual appearance of ventricular wall motion, wall thickening and changes in ventricular cavity size using the four‐chamber or short‐axis views (Videoclips S1 and S2). Although this qualitative assessment reflects an experience‐based, non‐quantitative impression rather than a definitive or reproducible measure of cardiac function, it is a valuable screening tool that may prompt further detailed assessment of ventricular function if abnormalities are suspected. Appropriate probe selection, depth and sector width help to maintain adequate spatial resolution. Gain settings should be optimized, as excessive gain may falsely thicken ventricular walls and obscure contractility, whereas too little gain can make the ventricles appear ‘akinetic’. Acute changes in fetal heart rate, as may occur with uterine pressure, can negatively impact two‐dimensional (2D) ultrasound and Doppler assessment of fetal cardiac function and should be avoided. The ventricles should be seen to contract symmetrically. Wall motion should be assessed for global abnormalities (Videoclip S3) and for segmental abnormalities. Segmental akinesia (lack of movement) or dyskinesia (dyssynchronous movement compared with other ventricular wall segments) seen in pathologies such as ventricular aneurysms and Ebstein's anomaly (Videoclips S4 and S5) should be excluded. Asymmetric motion could also suggest univentricular dysfunction, as seen in severe semilunar valve regurgitation or obstruction (Videoclips [Link], [Link], [Link]).

Although qualitative assessment is valuable and efficient, it is subjective, and its interpretation may vary among physicians. Developing experience through deliberate observation of fetal cardiac function in every sonographic assessment of the fetal heart, across a spectrum of heart disease and gestational ages from the late first trimester onward, increases reporting consistency and recognition of more subtle dysfunction. However, in fetuses at risk of cardiac dysfunction or with signs suggestive of cardiovascular compromise, including cardiomegaly, AV valve regurgitation or hydrops fetalis, a quantitative approach to cardiac function evaluation is warranted. Quantitative measures can be reliable and reproducible, but they too have limitations. Diminutive cardiac chambers, fetal movements and suboptimal acoustic windows compromise endomyocardial border definition, leading to measurement variability. As such, results of any measure should be interpreted in the context of the clinical scenario rather than taken in isolation.

It is also important to acknowledge that, in clinical practice, no single parameter is sufficient to assess and quantify fetal cardiac function comprehensively. Therefore, a multiparametric approach is recommended, combining qualitative assessment, as described above, with additional basic biometric assessments, such as the cardiothoracic ratio, chamber sizes (to derive shortening fraction (SF) or ejection fraction (EF)) and Doppler‐derived indices, such as AV inflow patterns and the myocardial performance index (MPI). More advanced techniques, such as tissue Doppler and deformation imaging, may provide additional information, but are not used routinely in all settings.

#### Ventricular shortening fraction (Figure 1)

M‐mode is an ultrasound technique that tracks tissue motion over time, capturing a single slice through the heart at high sampling rates. To measure ventricular SF using M‐mode, an axial four‐chamber or short‐axis 2D image of the fetal heart is acquired that clearly displays the anterior and posterior ventricular walls and septum in a horizontal orientation. Image depth and magnification should be adjusted so that the fetal heart occupies more than two‐thirds of the image sector, and a high‐resolution grayscale setting should be used to achieve high contrast and improved tracings. The M‐mode beam is positioned to intersect simultaneously the anterior and posterior ventricular walls and the ventricular septum. The beam should be at the level of the mitral valve papillary muscles, midway between the mitral valve annulus and the LV apex. Current ultrasound systems allow adjustment of the beam angle to optimize alignment perpendicular to the septum and the posterior wall, thereby capturing LV expansion and contraction. Moreover, using a dual‐screen display that captures both 2D and M‐mode images enables verification of the beam position. Once multiple cycles have been captured, using calipers, the end‐diastolic diameter (EDD) and end‐systolic diameter (ESD) of the RV and LV can be measured, and SF (%) can be calculated using the equation ((EDD − ESD)/EDD) × 100. Normal fetal LV‐SF and RV‐SF, independent of gestational age, are > 28% and > 24%, respectively^3^. In addition to assessing systolic function using SF, M‐mode can be used to confirm abnormal regional wall motion of the septum or free walls of the heart.

**Figure 1:**
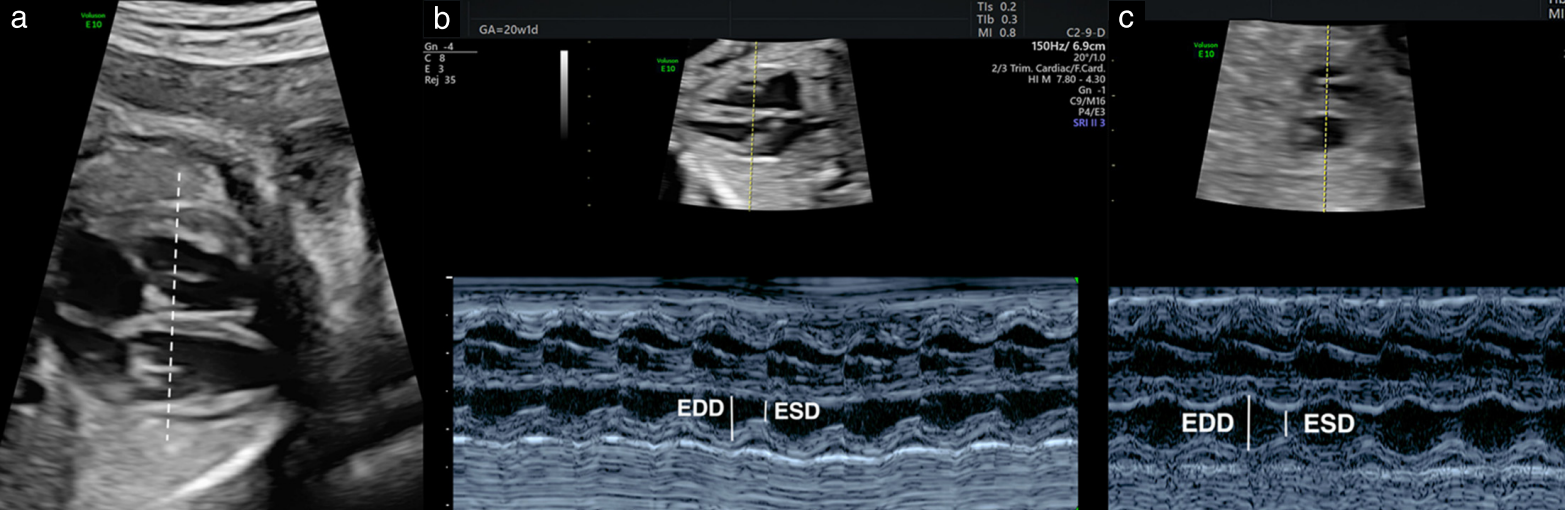
Assessment of ventricular shortening fraction (SF). (a) Optimized four‐chamber view with adjustment of two‐dimensional image depth and magnification so that the fetal heart occupies more than two‐thirds of the image sector. Plane of the M‐mode cursor passes through the right ventricular free wall, ventricular septum and left ventricular free wall perpendicular to their plane and just below the mitral valve annulus. (b) The four‐chamber M‐mode tracing acquired demonstrates multiple cardiac cycles. The end‐diastolic diameter (EDD) is measured as the largest diameter of the ventricle, shown here in the left ventricle, and the end‐systolic diameter (ESD) as the smallest diameter. SF (%) is calculated as ((EDD − ESD)/EDD) × 100. (c) From a four‐chamber view with the ventricular septum and free walls perpendicular to the ultrasound beam, the transducer is rotated 90°, and an M‐mode tracing through the ventricular short axis at the level of the ventricular papillary muscles can also be used to assess SF. Dashed line indicates M‐mode cursor.

#### Annular plane systolic excursion (Figure 2 *)*

**Figure 2:**
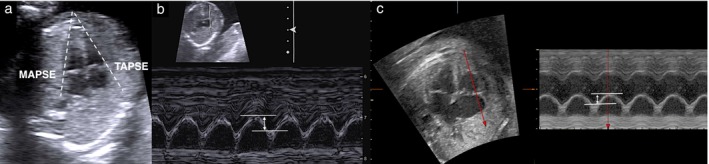
Assessment of tricuspid (TAPSE) and mitral (MAPSE) annular plane systolic excursion. (a) TAPSE and MAPSE are obtained from an apical four‐chamber view with the apex oriented toward the transducer, and the M‐mode cursor (dashed lines) placed from the ventricular apex to the lateral aspect of each valve. (b) Longitudinal annular motion is displayed by M‐mode, and TAPSE and MAPSE can be calculated as the distance that the annulus moves between systole and diastole (double‐headed arrow). (c) Example of spatiotemporal image correlation (STIC) (red arrow) used to assess TAPSE in a 26‐week fetus with hydrops. Despite suboptimal fetal position for M‐mode cursor alignment, STIC permits maneuvering of the image to optimize cursor placement.

M‐mode can also be used to assess the tricuspid annular plane systolic excursion (TAPSE) and mitral annular plane systolic excursion (MAPSE), which are also measures of systolic function. TAPSE and MAPSE are obtained by placing the M‐mode cursor from the ventricular apex through the lateral tricuspid or mitral annulus, respectively, in order to track the longitudinal motion of the AV valve annulus throughout the cardiac cycle. TAPSE and MAPSE are measured as the distance between the systolic and diastolic positions of the tricuspid and mitral valve lateral annulus, respectively, as shown in Figure 2. The magnitude of movement of the valve annular plane on the M‐mode tracings from its diastolic to systolic position correlates with ventricular systolic function, which increases with gestational age^4^. Given that RV contraction occurs predominantly along the long axis, whereas LV contraction occurs more along the short axis^5^, this parameter is likely more representative of RV function.

#### Ejection fraction and fractional area change (Figure 3)

EF estimates the percentage of the ventricular end‐diastolic volume (EDV) ejected by the ventricle with each heartbeat. Although three‐dimensional (3D) imaging may be more accurate, EF can be measured from 2D ultrasound images and speckle‐tracking echocardiography (STE) using the Simpson method^6^, which calculates ventricular volume by assuming that the ventricle has an ellipsoid shape. To measure EF, an image is acquired in the apical four‐chamber view at a high frame rate (> 60 fps), with the ventricular apex at the center of the imaging sector and the AV valve as horizontal as possible, avoiding foreshortening of the cavity. For EDV, an end‐diastolic frame before AV valve closure is selected, which is when the ventricular cavity is at its largest. The endocardial border is then traced manually, starting and ending on either side of the AV valve insertion points. Similarly, a systolic measurement for end‐systolic volume (ESV) is performed to obtain the smallest ventricular cavity dimension, just before AV valve opening. The LV is divided into stacked ‘disks’ of predetermined volumes in end‐diastole and end‐systole, and software can be used to derive EDV and ESV accordingly. The LV‐EF (%) is calculated as ((LV‐EDV − LV‐ESV)/LV‐EDV) × 100. The mean fetal LV‐EF measured using the single‐plane Simpson's method ranges from 60% to 70% in the second and third trimesters and should not be < 55%^6^. Comparable values have also been reported for both ventricles using the biplane Simpson's method, which combines four‐ and two‐chamber views^7^.

**Figure 3:**
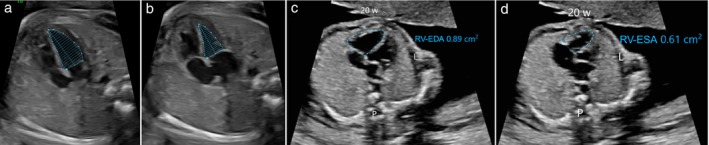
Assessment of ventricular ejection fraction (EF) and fractional area change (FAC). (a) EF is measured from a high frame rate (> 60 fps) apical four‐chamber view, avoiding ventricular foreshortening. Ventricular cavity measurement using Phillips Intelli Space Cardiovascular software (Phillips Healthcare, Andover, MA, USA) to calculate end‐diastolic volume (EDV) by tracing the ventricular endocardial border at end‐diastole (largest cavity, just before atrioventricular (AV) valve closure) is shown. (b) For measurement of end‐systolic volume (ESV), the frame with the smallest ventricular cavity, just before AV valve opening, is selected and the endocardial border traced from one side to the other of the AV valve annulus. EF is then calculated using the equation (EDV − ESV)/EDV. (c) Measurement of the right ventricular (RV) end‐diastolic area (EDA) using the image with the largest RV cavity is shown. (d) To obtain the RV end‐systolic area (ESA), RV is measured when the cavity is smallest. FAC (%) is then calculated as ((RV‐EDA − RV‐ESA)/RV‐EDA) × 100. For the 20‐week (w) fetus shown in (c,d), the FAC would be ((0.89−0.61)/0.89) × 100 = 31%. L, left; P, posterior.

Although EF can be calculated for both ventricles, it is less accurate for the RV because of its non‐ellipsoid shape, a limitation that also applies to many single‐ventricle hearts. Fractional area change (FAC), which assesses how much the ventricular cavity decreases in systole using the end‐diastolic area and end‐systolic area of a ventricle measured from a four‐chamber image using the technique described above, can be applied to either ventricle, but is a preferred measure for the RV after birth. FAC (%) is calculated as ((end‐diastolic area − end‐systolic area)/end‐diastolic area) × 100. LV‐FAC should be greater than RV‐FAC^8^, and both decrease with gestational age, at least until the early third trimester^8^, ^9^. Using 2D ultrasound imaging, published normative data demonstrate that the mean RV‐FAC is 36% at 18 weeks' gestation, decreasing to 29% by term^9^.

These 2D area‐ and volume‐based methods provide more objective measures of biventricular systolic function, are reproducible, are sensitive to early dysfunction and allow for serial monitoring. FAC has shown a strong correlation with longitudinal and transverse FS, is angle‐independent and correlates with other parameters of biventricular function^10^; however, there is no gold standard for assessment of fetal biventricular function against which to compare. Limitations of these methods include difficulty defining the endocardial border in low‐resolution images and load dependency, which can elevate FAC and EF in high‐output lesions even if myocardial dysfunction is present.

### Assessing ventricular diastolic function (Figure 4)

Diastolic function is assessed by evaluating pulsed‐wave (PW) Doppler flow patterns of the AV valves and systemic and pulmonary veins. Fetuses normally exhibit gestational‐age‐related ventricular relaxation patterns, characterized by higher A‐wave (atrial contraction) than E‐wave (early diastolic filling across the mitral valve) velocities. However, even with this pattern, a biphasic inflow pattern is typically observed beyond 10 weeks. The presence of fused A‐ and E‐waves can make interpreting the E/A ratio challenging, but the ventricular filling duration should still be normal. The isovolumetric relaxation time, as well as systemic and pulmonary venous, ductus venosus and umbilical vein flow profiles, are all important for assessing ventricular and central filling pressures. With ventricular dysfunction in pathologies such as cardiomyopathy^11^, twin‐to‐twin transfusion^12^, ^13^ and certain structural heart lesions associated with heart failure, ventricular inflow Doppler waveforms become monophasic and shortened in duration. Conversely, cardiac compression from intrathoracic masses and effusions is associated with a biphasic flow pattern with a dominant E‐wave^14^. When ventricular filling pressures are significantly elevated, right atrial pressure increases, which results in loss of early diastolic forward flow and increased A‐wave reversal in the inferior vena cava and hepatic veins, A‐wave reversal in the ductus venosus and eventual umbilical venous pulsations. Non‐immune hydrops, characterized by the development of extravascular fluid collection and skin edema, often ensues, and acute fetal demise due to hypoxemia may occur.

**Figure 4:**
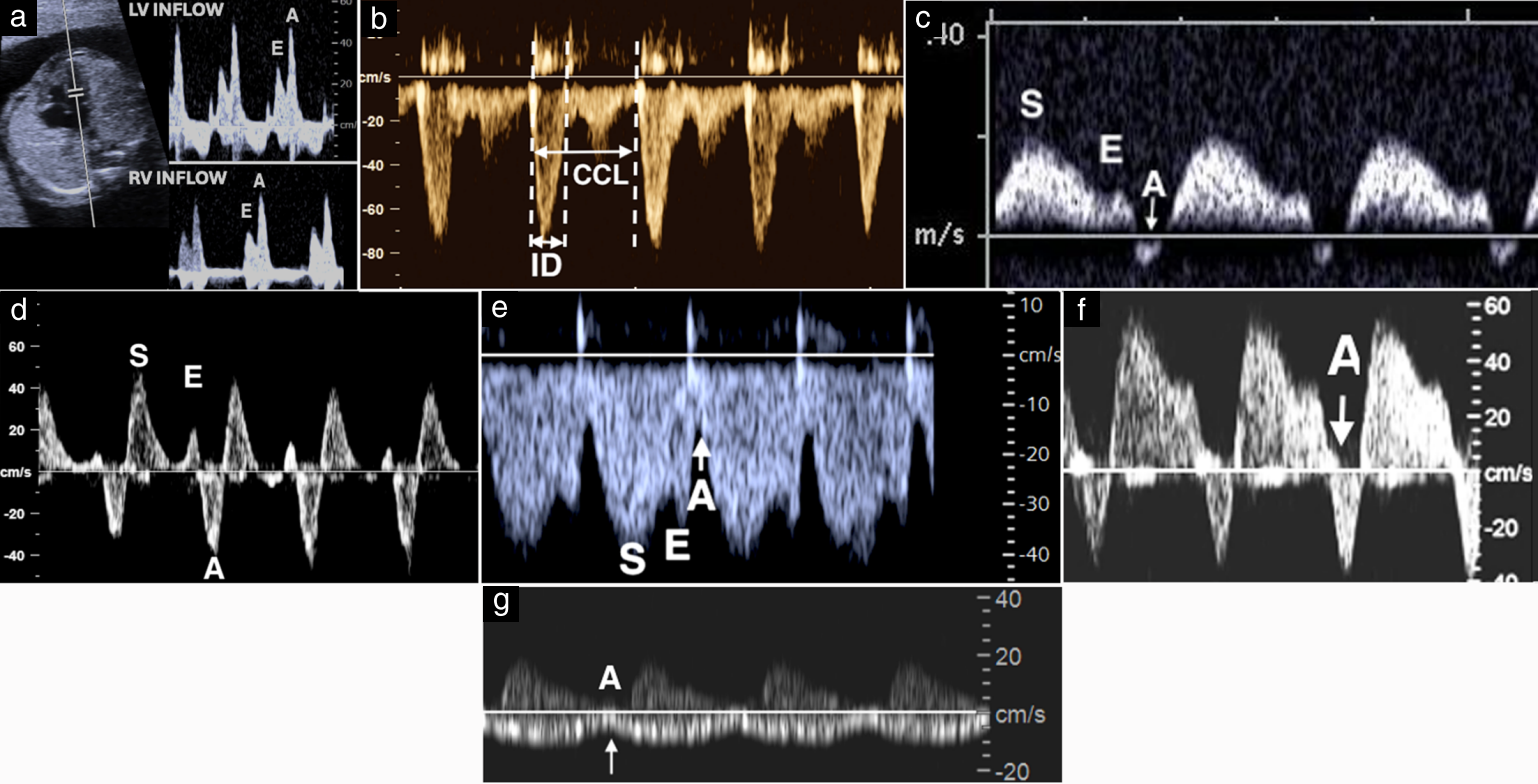
Assessment of diastolic function through pulsed‐wave Doppler interrogation. (a) Normal left (LV) and right (RV) ventricular Doppler inflow acquisition requires alignment of the Doppler sample volume parallel to flow (< 20°), at or just below the atrioventricular valve annulus, as demonstrated for the LV (left panel). Normal Doppler flow profile sampled through the mitral (left) and tricuspid (right) valves demonstrates the usual biphasic flow pattern with E‐ (early diastole) and A‐ (atrial contraction) waves and a duration of nearly half the cardiac cycle length. (b) Uniphasic ventricular inflow (ID, inflow duration) of very short duration (during atrial systole) relative to the cardiac cycle length (CCL) is shown in a fetus with dilated cardiomyopathy. (c) Normal inferior vena cava and hepatic venous Doppler profile demonstrates three phases: forward flow in systole (S), forward flow in early diastole (E) and short velocity A‐wave reversal during atrial contraction (A). (d) With increased ventricular and atrial filling pressures there is loss of flow in early diastole and progressive A‐wave reversal (velocity and duration). Eventually flow may become bidirectional. (e) The normal ductus venosus flow profile has the same three phases as seen for the systemic veins; however, during atrial systole there is a reduction in forward flow velocity and no flow reversal should be present. (f) With increasing central venous pressures due to poor function, there is eventual reversal of flow in the ductus venous during atrial systole. (g) Umbilical venous Doppler demonstrates abnormal pulsatility with reduction or cessation of flow in atrial systole. Normally, there is low velocity non‐phasic flow or undulating flow with fetal breathing.

To assess diastolic function, a PW Doppler sample volume is placed at the tips of the AV valve leaflets, ensuring alignment of the Doppler beam parallel to blood flow. With the dual‐display setting, the Doppler cursor position and the waveform can be displayed simultaneously. E‐ and A‐wave velocities, E/A ratio and inflow duration should be assessed and compared to expected gestational‐age‐based reference ranges^3^. PW Doppler sampling of the inferior vena cava and hepatic veins, with the Doppler beam aligned parallel to flow, allows assessment of increasing A‐wave reversal as an indicator of increased downstream filling pressures. Finally, PW Doppler sampling of the ductus venosus and umbilical vein should be performed to exclude absent or reversed A‐wave as a marker for increased filling pressure and cardiac compromise. Pulmonary vein flow patterns should similarly be assessed for A‐wave reversal, reflecting LV diastolic dysfunction.

### Cardiac output assessment (Figure 5)

Fetal combined cardiac output (CCO) estimates the amount of blood volume ejected from the heart per minute. It is measured by combining biventricular outputs expressed as mL/min/kg of estimated fetal weight, or by calculating the *Z*‐score from normative gestational‐age data. Individual LV and RV outputs can be estimated as follows: the semilunar valve cross‐sectional area (CSA) is calculated from the valve diameter (π × (diameter/2)^2^), and then multiplied by the velocity time integral (VTI) traced from the semilunar valve Doppler waveform, to give the ventricular stroke volume. This is multiplied by the fetal heart rate to give the ventricular output.

**Figure 5:**
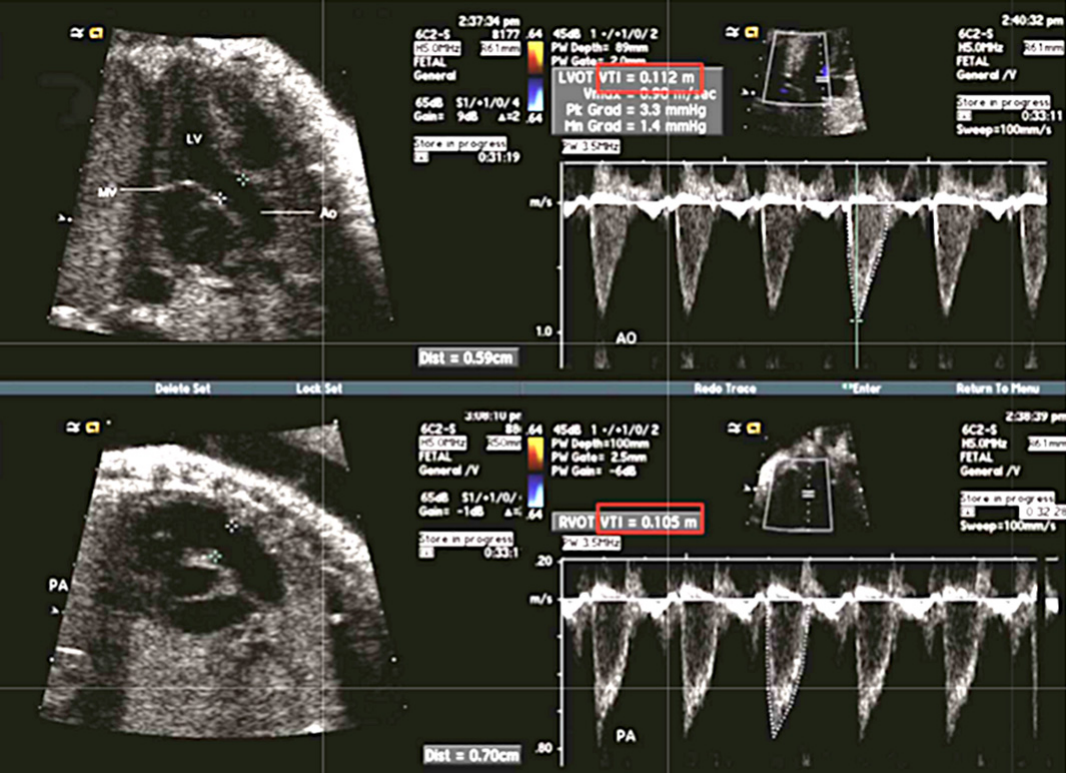
Calculation of fetal left and right ventricular outputs. Still‐frame 2D ultrasound images and Doppler spectra demonstrating the Doppler‐based method of calculating fetal ventricular stroke volumes and outputs. Aortic (top left) and pulmonary (bottom left) valve annuli are measured in systole in as close to an axial plane as possible. The cross sectional area (CSA, in cm) of the valve is then calculated using the equation π × (valve diameter/2)^2^. Pulsed‐wave Doppler interrogation through the aortic (top right) and pulmonary (bottom right) valves with the cursor aligned parallel to flow (and no angle correction) provides a spectral tracing for each valve, which are then planimetered to generate the velocity time integral (VTI) in cm. The ventricular stroke volume (SV) is calculated using CSA × VTI (in cm^3^ or mL) and ventricular output is calculated using SV × fetal heart rate. By combining the ventricular outputs (usually measured in units of mL/min and indexed by estimated fetal weight (mL/kg/min)), the combined cardiac output can be derived. Ao, aorta; LV, left ventricle; LVOT, left ventricular outflow tract; MV, mitral valve; PA, pulmonary artery; RVOT, right ventricular outflow tract.

To obtain the most accurate ventricular output measurement, the PW Doppler tracing should be acquired with the Doppler beam positioned parallel to flow, with an insonation angle as close as possible to 0° and without angle correction, sampling two to three cardiac cycles. Valve diameters in midsystole should be obtained in the axial plane. Precise valve measurements are vital because any error is magnified. Reference ranges for CCO have been published previously^15^, ^16^, and an online *Z*‐score generator is available (http://fetal.parameterz.com/). CCO estimation is useful in assessing fetal hydrops of undefined etiology and tracking high‐output cardiac lesions, such as fetal anemia, vascular tumors, vascular arteriovenous malformations, extrahepatic insertion of the umbilical vein and the ‘pump’ twin (i.e. the structurally normal twin) in twin reversed arterial perfusion (TRAP) sequence. A CCO of > 600 mL/min/kg is associated with increased risk of adverse outcomes in high‐cardiac‐output pathologies^17^, ^18^. CCO assessment may also be useful for tracking lesions associated with low cardiac output.

### Myocardial performance index (Figure 6)

MPI, also referred to as the Tei index, is the ratio of the sum of the isovolumetric contraction time (ICT) and isovolumetric relaxation time (IRT) to the ejection time, and it increases with declining global function^19^.

**Figure 6:**
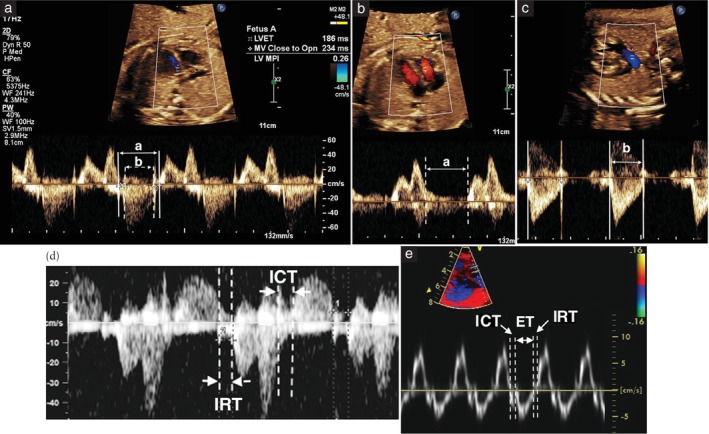
Assessment of myocardial performance index (MPI). (a) Left ventricular (LV) inflow‐outflow Doppler waveform with a sample gate placed within the LV in a location where both inflow (above baseline) and outflow (below baseline) signals are captured simultaneously, with measurement of ‘a’ (offset of one inflow to onset of next inflow) and ‘b’ (ejection time (ET)). Note the ‘valve clicks’, which can guide measurement. The MPI is calculated using the equation (a − b)/b. (b,c) Calculation of MPI for the right ventricle (RV) requires separate inflow and outflow Doppler signals. (b) From inflow tracings, ‘a’ can be measured from the offset of one inflow to the onset of the other. (c) From the outflow tracings, ‘b’ is measured as the ET. (d) The numerator in the MPI equation (a − b) equates to the sum of the isovolumetric relaxation time (IRT, from offset of outflow to onset of inflow) and isovolumetric contraction time (ICT, from offset of inflow to onset of outflow), as measured in this LV inflow‐outflow Doppler waveform. (e) MPI can also be assessed using tissue Doppler for both RV and LV. Similarly, valve clicks are helpful to guide measurement. Unlike with pulsed‐wave Doppler, a simultaneous inflow‐outflow tissue Doppler signal can be used to measure RV‐MPI. A major disadvantage is lack of availability of tissue Doppler imaging software/resources on many ultrasound systems.

To measure the LV‐MPI, the PW Doppler cursor should be placed within the LV cavity, at a location where both inflow and outflow signals are captured simultaneously, which is feasible given the morphological proximity of the LV inflow and outflow tracts. To improve accuracy, time intervals can be measured using the bright thin vertical spike on the Doppler tracing representing the leaflet motion with valve closure (‘valve clicks’), and the sweep speed can be increased to broaden tracings over a cardiac cycle. RV‐MPI requires PW Doppler assessment of the tricuspid inflow and pulmonary outflow in succession, as the RV inflow and outflow tracts lie in different imaging planes. The fetal heart rate should not differ by more than 10 bpm between image acquisitions to ensure measurement accuracy.

The MPI combines systolic and diastolic dysfunction into a single ‘global’ measure of ventricular function. Normative reference ranges for RV‐MPI and LV‐MPI have been published previously^20^, ^21^. In a previous systematic review and meta‐analysis, mean MPI was 0.40 (95% CI, 0.374–0.426) at 12 weeks, increasing to 0.58 (95% CI, 0.533–0.637) by term, in normal pregnancies. MPI can also be measured using tissue Doppler imaging in a manner similar to PW Doppler assessment, as described above. Tissue Doppler‐derived MPI values have also been reported to increase with gestational age, with a greater increase for the LV than for the RV^22^.

MPI increases with reduced contractility, impaired relaxation and/or increased afterload of the ventricles. Importantly, it is independent of ventricular geometry and is highly reproducible, making it suitable for serial assessment. Additionally, MPI detects early signs of myocardial decompensation, as observed in twin‐to‐twin transfusion syndrome cardiomyopathy^12^, ^13^. However, MPI measurements are highly operator‐dependent, and small measurement errors can lead to significant differences in MPI. Developing standardized laboratory protocols for image acquisition and MPI measurement helps to provide consistency among team members and improves MPI reproducibility. Furthermore, MPI assessment requires a normal cardiac rhythm. It is afterload‐dependent, in that it increases in cases of outflow obstruction, ductal constriction and increased placental resistance, even when myocardial contractility is normal. If the MPI is abnormal, evaluation of the IRT and ICT (Figure 6d) can further elucidate the nature of the dysfunction.

### Experimental and emerging modalities

Advanced and automated cardiac function tools, including deformation imaging, ventricular dyssynchrony evaluation^23^, tissue Doppler imaging and 3D/four‐dimensional ventricular volume acquisition, are gaining interest amongst fetal cardiology and maternal–fetal medicine providers assessing fetal cardiac function, but such advanced modalities remain uncommon in routine practice. This is in part due to their limited availability across all ultrasound systems, lack of validation for some and untested prognostic value for others. Most are beyond the scope of this review.

#### Cardiac deformation and strain imaging (Figure 7)

Cardiac deformation is defined as the longitudinal and circumferential myocardial shortening and lengthening, as well as radial thickening and thinning, that occur during systolic contraction and diastolic relaxation. Deformation can be quantified using STE, which tracks natural acoustic myocardial ‘speckles’ from frame to frame. Measures of deformation that are commonly used include global systolic strain (change in myocardial length from end‐diastole to end‐systole) and systolic strain rate (the rate of change in length over time).

**Figure 7:**
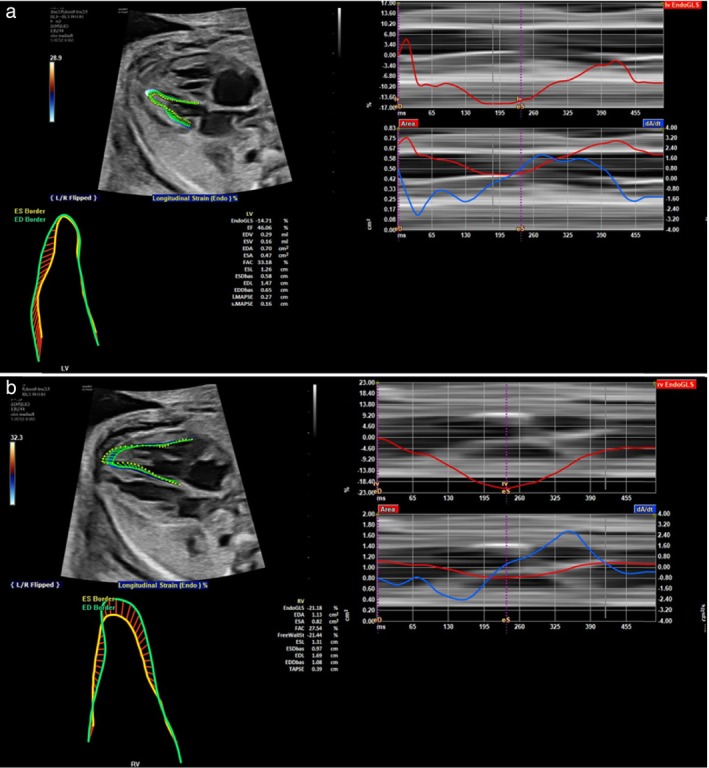
Assessment of ventricular strain. (a) Fetal left ventricular (LV) apical four‐chamber strain is derived from speckle‐tracking echocardiography using GE EchoPAC/Voluson integrated strain package. The LV endocardial border is planimetered in an apical four‐chamber view, and the myocardial ‘speckles’ are then tracked throughout systole and diastole with generation of tracings and measurement of strain and strain rate. The graph shown is a strain curve, demonstrating smooth systolic deformation with normal peak systolic strain followed by diastolic relaxation. Importantly, assessment of strain requires high frame rates to generate accurate measures and may differ between different software. (b) Similarly, the right ventricular strain and strain rate can be assessed. ED Border, end‐diastolic endocardial border; ES Border, end‐systolic endocardial border.

To obtain global longitudinal strain (GLS), an apical four‐chamber view is used in which the entire ventricle is visible without foreshortening. Images are optimized to achieve high frame rates (> 80 fps, ideally). Image acquisition should include three to five cardiac cycles without significant fetal motion to allow accurate GLS analysis. The endocardial border is defined once the user selects a few manual points (e.g. the AV valve annulus and apex). The border delineation may be automated or semiautomated using border‐detection algorithms in different software. Depending on the software, the user may also need to define the epicardium and adjust the myocardial thickness to ensure that only the myocardium is included. The software will then track myocardial motion throughout the cardiac cycle and produce GLS values. Manual confirmation that the tracking is concordant with myocardial movement is required.

In general, a GLS of −20% to −25% is considered normal^10^, ^24^, and less negative values indicate ventricular dysfunction. Deformation is independent of chamber geometry, although not totally angle‐independent due to resolution asymmetry and section shape^25^.

Although fetal STE is a promising technology, concerns remain about its reproducibility, especially when it is performed retrospectively^26^. Strain values can vary across vendors, a challenge for multicenter research and clinical practice^27^. Prospectively analyzed datasets may be more reproducible. Performing strain analysis at native frame rates rather than compressing for storage is advantageous.

#### 3D volumes

3D echocardiography allows direct volumetric assessment of ventricular size and function without relying on geometric assumptions and can be used to calculate ventricular EF more accurately than is possible using the 2D methods described above. 3D volumes can be measured using the spatiotemporal image correlation (STIC) method, which acquires a volume dataset over multiple cardiac cycles and reconstructs a single cycle for analysis^28^, ^29^. Image acquisition is performed when fetal and maternal movements are minimal. Once acquired, analysis is completed using automated segmentation of the ventricular cavity, if such software is available on the ultrasound system, or using semiautomated or manual segmentation, depending on the available resources.

Given its independence from geometric assumptions, 3D imaging may be better for functional assessment of the RV or a single ventricle. However, the accuracy of this technique is highly operator‐dependent and can be significantly time‐consuming if offline analysis is required. Furthermore, it is very sensitive to image resolution, fetal motion and heart rhythm abnormalities. Due to these limitations, intraobserver variability is moderate. Currently, this technique is used primarily as a research or adjunct tool.

### Cardiovascular profile score (Table 2)

The cardiovascular profile (CVP) score^30^ incorporates five categories of ultrasound marker, each with a 2‐point score, with a total maximum score of 10 points, as listed in Table 2. These five categories include: evidence of fetal hydrops; cardiomegaly (cardiothoracic area ratio); abnormal myocardial function based on various measures as described above; abnormal umbilical arterial Doppler findings; and abnormal venous Doppler findings. While these measures are components of a complete fetal echocardiogram, deriving a CVP score can provide a global assessment of the severity of heart failure and fetal–placental compromise, enabling serial monitoring and decision‐making. The CVP score has a high prognostic value in high‐risk fetal congenital heart disease (CHD)^30^, ^31^, ^32^ and in intrauterine growth restriction without CHD^33^. A normal CVP score is 10/10, and the presence of an abnormality reduces the score proportionally. A CVP of ≤ 7 indicates a high risk of either *in‐utero* death or postnatal mortality.

**Table 2:** Cardiovascular profile score

Category	2 points	1 point	0 points
Hydrops	None	Ascites or pericardial or pleural effusion	Fluid in > 2 cavities or skin edema
Heart size (HA/CA)	> 0.2 and ≤ 0.35	> 0.35–0.5	≤ 0.2 or > 0.5
Cardiac function	Normal MV and TV, biphasic diastolic filling, LV‐SF or RV‐SF ≥ 0.28	Holosystolic TR or LV‐SF or RV‐SF < 0.28	Holosystolic MR or TR dP/dt < 400, monophasic diastolic filling
UA Doppler	Normal	AEDV	REDV
Venous Doppler	Normal	DV atrial reversal	UV pulsations

Table adapted from Wieczorek *et al*.^30^. AEDV, absent end‐diastolic velocity; DV, ductus venosus; HA/CA, heart‐to‐chest area ratio; LV, left ventricle; MR, mitral valve regurgitation; MV, mitral valve; REDV, reversed end‐diastolic velocity; RV, right ventricle; SF, shortening fraction; TR, tricuspid valve regurgitation; TR dP/dt, change in pressure over time of TR jet; TV, tricuspid valve; UA, umbilical artery; UV, umbilical vein.

### Grading of fetal ventricular dysfunction

At present, there is no universally accepted grading system for fetal cardiac dysfunction, and the interpretation is based on a multiparametric approach integrating qualitative and quantitative findings, as described above. The CVP score may provide additional resources, allowing serial assessment and monitoring of changes in function or hemodynamic effects of reduced function over time. A pragmatic framework may be considered, whereby mild dysfunction would suggest subtle or isolated cardiac abnormalities, and moderate dysfunction would indicate more consistent evidence across multiple parameters. In contrast, severe dysfunction would be associated with significant hemodynamic compromise and a reduced CVP.

### Reporting

Reporting on fetal cardiac function should include a description of global biventricular systolic function, AV valve inflow patterns and any valve regurgitation, as well as evidence of venous congestion, chamber enlargement or global cardiomegaly, effusions, frank fetal hydrops and the CVP score, as applicable.

### Serial assessment

Whether there is a risk or evidence of ventricular dysfunction, serial assessment is paramount. Ventricular dysfunction is often a progressive process that, depending on the underlying etiology, may initially involve either systolic or diastolic impairment, or present with concomitant abnormalities of both. Using multiple qualitative and quantitative imaging and measurement techniques and serial assessments, depending on the predicted progression of disease, ensures that early dysfunction is detected promptly. The finding of ventricular dysfunction should guide future surveillance and should be integrated into delivery and acute neonatal care planning.

## CONCLUSION

Assessment of fetal ventricular function is an integral component of fetal cardiac evaluation. Although qualitative assessment is sufficient for most routine fetal echocardiograms, fetuses at risk for ventricular dysfunction should undergo more comprehensive and quantitative assessment of ventricular function. Amongst the various quantitative methods described herein, combining 2D‐based measures (such as ventricular SF, EF and FAC) with Doppler‐based parameters (such as ventricular inflow patterns, MPI or TAPSE and MAPSE) may improve the accuracy of global assessment, while also providing a baseline for serial monitoring. Emerging modalities (such as 3D‐STIC volume analysis and strain imaging) are promising, but are not yet used routinely in clinical practice. In addition to quantitative measures of ventricular function, calculation of the CVP score provides further global assessment of fetal heart failure and placental compromise, facilitating serial monitoring and informing decisions regarding timing for delivery. Use of standardized imaging and measurement protocols and workflows within individual centers is recommended to improve consistency, accuracy and reproducibility of fetal ventricular function assessment.

## Supporting information


**Videoclip S1** Grayscale ultrasound four‐chamber cine loop, showing normal biventricular size, wall thickness and systolic function. L, left; P, posterior; w, weeks.

**Videoclip S2** Grayscale ultrasound short‐axis cine loop, showing normal biventricular size, wall thickness and systolic function. w, weeks.

**Videoclip S3** Grayscale ultrasound four‐chamber cine loop, showing cardiomegaly, biventricular dilation and dysfunction. L, left; P, posterior; w, weeks.

**Videoclip S4** Grayscale ultrasound four‐chamber cine loop, showing a large right ventricular aneurysm (arrow) with dyssynchronous movement. L, left; P, posterior; w, weeks.

**Videoclip S5** Grayscale ultrasound four‐chamber cine loop, showing dyssynchronous septal motion (arrow) in a fetus with Ebstein's anomaly. L, left; P, posterior; w, weeks.

**Videoclip S6** Grayscale ultrasound four‐chamber cine loop, showing enlarged right atrium and right ventricle with right ventricular dysfunction (arrow) in a fetus with tetralogy of Fallot with absent pulmonary valve. Note how differently the right and left ventricles contract. L, left; P, posterior; w, weeks.

**Videoclip S7** Grayscale ultrasound four‐chamber cine loop, showing enlarged left ventricle with severe dysfunction and endocardial fibroelastosis (arrow) in a fetus with critical aortic stenosis. Right ventricular systolic function is normal. L, left; P, posterior; w, weeks.

**Videoclip S8** Grayscale ultrasound four‐chamber cine loop, showing mildly dilated left ventricle with mild to moderate left ventricular free wall dysfunction and endocardial fibroelastosis involving the mitral valve chordal apparatus (arrow) in a fetus with severe aortic stenosis. Fetus has dextrocardia but visceroatrial situs solitus and normal cardiac segments and alignments. L, left; P, posterior; w, weeks.

## Data Availability

Data sharing not applicable to this article as no datasets were generated or analysed during the current study.
